# Integrative transcriptomic and proteomic analysis reveals CD9/ITGA4/PI3K‐Akt axis mediates trabecular meshwork cell apoptosis in human glaucoma

**DOI:** 10.1111/jcmm.14792

**Published:** 2019-11-03

**Authors:** Junwei Yan, Xuejiao Yang, Xuefei Jiao, Xian Yang, Mingjin Guo, Yunqing Chen, Lu Zhan, Wenshi Chen

**Affiliations:** ^1^ Department of Vascular Surgery The Affiliated Hospital of Qingdao University Qingdao China; ^2^ Department of Ophthalmology The Affiliated Hospital of Qingdao University Qingdao China; ^3^ Department of Pathology The Affiliated Hospital of Qingdao University Qingdao China

**Keywords:** apoptosis, CD9, glaucoma, transcriptomics and proteomics analysis

## Abstract

Glaucoma has been the leading cause of irreversible blindness worldwide. High intraocular pressure (IOP) is a high‐risk factor of glaucoma, repression of which has been the important treatment of glaucoma in clinic. Trabecular meshwork is crucial for maintaining IOP in aqueous humour out‐flow system. It is urgent to reveal the molecular mechanism of trabecular meshwork in glaucoma. Previous studies found that some pathways were related to glaucoma, such as extracellular matrix (ECM)‐receptor interaction, phosphatidylinositol 3‐kinase (PI3K)‐protein kinase B (Akt) and apoptosis. To identify novel molecules in glaucoma, we performed high‐throughput transcriptome and proteome analysis to immortal human trabecular meshwork cells (iHTM) and glaucomatous human trabecular meshwork cells (GTM_3_), respectively. Twenty‐six up‐regulated genes/proteins and 59 down‐regulated genes/proteins were identified as the high‐risk factors based on differential analysis, including some known factors of glaucoma. Furthermore, a glaucoma‐related protein‐protein interaction (PPI) network was constructed for investigating the function roles of risk factors. Some genes were identified as potential regulator in the pathogenesis of glaucoma based on the topology analysis and module analysis to the network. Importantly, we identified and demonstrated that CD9 played key roles in glaucoma by biological experiment. CD9 is down‐regulated in glaucoma, overexpression of CD9 can active integrin α4 (ITGA4), PI3K and Akt, which lead to the decreased apoptosis and attenuate glaucoma. All these results provide a novel molecular therapy of glaucoma.

## 1 INTRODUCTION

Glaucoma has been the leading cause of irreversible blindness worldwide and notably.^1^, ^2^ An increased intraocular pressure (IOP) phenotype is often followed with primary open‐angle glaucoma (POAG) and is caused by excessive resistance to the out‐flow of the aqueous humour via multiple downstream pathways.^3^, ^4^ Trabecular meshwork (TM) is a cavernous‐like filtrating structure that located at anterior chamber angle of human eyeball, and it is composed of cross‐liked multi‐laminar and spindle meshwork tissues. Previous studies demonstrated that trabecular meshwork cells in aqueous humour out‐flow system were functioned as detector and responder to mechanical forces, adapting their physiology to maintain cellular function.^5^ Thus, the cellular state of trabecular meshwork is highly related to IOP and determines the pathological processes of POAG. Although the pathological mechanism of glaucoma is unclear, it is reported that extracellular matrix (ECM), oxidative stress, TGFβ and apoptosis signal pathways are crucial regulators of glaucoma.^6^, ^7^, ^8^, ^9^

The potential role of ECM properties on aqueous humour out‐flow system constitutes the out‐flow path within the trabecular meshwork. Increasing evidences demonstrated that reactive oxygen species (ROS), which can lead to oxidative stress, is a crucial regulator in the pathogenesis of POAG, including resistance to aqueous humour out‐flow, alteration of superoxide dismutase‐catalase and glutathione pathway activity and intraocular pressure.^10^ Apoptosis is also considered as a key regulator in the pathological processes of POAG. In addition, the PI3K‐Akt signalling pathway is a key intracellular signalling transduction pathway, which can promote cell proliferation, repress apoptosis and induce angiogenesis by activating its multiple downstream factors. And studies found that MALAT1 can regulate apoptosis activity of retinal ganglion cells through PI3K/Akt signal in glaucoma.^11^

With the rapid development of high‐throughput techniques, more and more researches were investigated by integrating multiple omics. For instance, Li et al performed a combined transcriptomic and proteomic pipeline to investigate the venom composition of jellyfish *C nozakii* and identified 174 potential toxin proteins, which was helpful to comprehensive understanding of the venom composition and to identify novel methods for jellyfish sting.^12^ Cheng et al revealed that the Pde10a is elevated in miR‐137 knockout mice by using transcriptomic and proteomic analysis. miR‐137 was demonstrated to play important roles in the processes of postnatal neurodevelopment. Dysfunction of miR‐137 could lead to neuropsychiatric disorders in humans.^13^ Furthermore, Lee et al integrated transcriptomic and cell surface proteomic data identified new immune‐based therapy targets in subtypes of advanced prostate cancer, such as FXYD3 and CEACAM5.^14^ All these findings suggested us to use a combined multiple omic pipeline to identify novel regulatory axis that functions in POAG.

**Figure 1:**
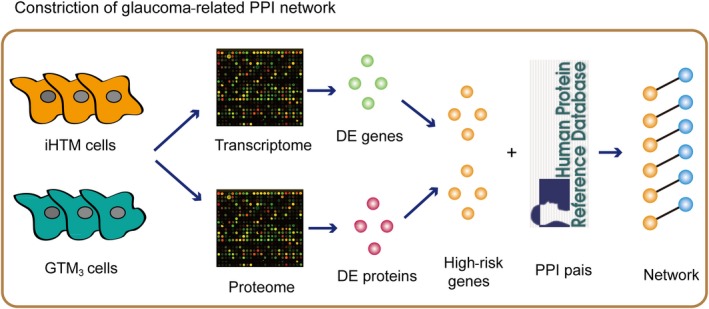
The pipeline for construction of glaucoma‐related PPI network. First, we intersected all differentially expressed (DE) genes and proteins. Second, we mapped all these risk genes into the HPRD network and extracted the risk gene associated subnetwork. Third, all risk gene associated pairs were merged into the glaucoma‐related PPI network

In this study, we performed transcriptomic and proteomic data to the immortalized normal human trabecular meshwork cells (iHTM) and glaucomatous human trabecular meshwork cells (GTM_3_) for identifying novel molecular that function in glaucoma. As a result, 26 up‐regulated genes/proteins and 59 down‐regulated genes/proteins are considered as high‐risk factors of glaucoma, based on the strict threshold of differentially expression analysis. Results showed these genes were high related to PI3K‐Akt, focal adhesion, endocytosis and ECM‐receptor interaction. A glaucoma‐related protein‐protein interaction (PPI) network was constructed (Figure 1), after topology analysis and module analysis to the network, suggesting some genes were potential regulator in the pathogenesis of glaucoma. Importantly, we identified and demonstrated that CD9 played key roles in glaucoma by biological experiment. CD9 is down‐regulated in glaucoma, overexpression of CD9 can active integrin α4 (ITGA4), PI3K and Akt, which lead to the decreased apoptosis of TM cell and maintained the TM cell identity. Knock‐down of CD9 showed the reverse results. Furthermore, rescue experiment validated that CD9/ITGA4/PI3K‐Akt axis mediated TM cell apoptosis in glaucoma. All these results shed new light on the clinical therapy of glaucoma.

## 2 MATERIALS AND METHODS

### 2.1 Cell culture and transfection

iHTM was kindly provided by Dr Vincent Raymond (Laboratory of Ocular Genetics and Genomics) and GTM_3_ was obtained as a gift from Prof. Yuhao Peng (glaucoma research; Alcon Laboratory). They were isolated from the trabecular meshwork of a normal and a primary open‐angle glaucoma patient respectively, and then, they were transfected with an origin defective mutant of SV40 virus. In addition, we also performed experimental data to validate the TM cell identity. Results showed the TM cell markers were high expressed in iHTM cells and GTM_3_ cells (Figure S2). Cells were cultured in Medium of Nutrient Mixture F‐12 (DMEM/F12; Gibco, Invitrogen) that contained 15% fetal bovine serum (FBS; Gibco, Invitrogen) at 37°C and 5% CO_2_. CD9 vector generation was described in previous studies.^40^ GTM_3_ cells were transfected with pcDNA3.1/CD9 wild‐type construct using X‐tremeGENE reagent (Roche) following the manufacturer's instructions. After transfection, GTM_3_ cells were cultured 24 hours for further experiments. CD9 and ITGA4 siRNA that purchased from GenePharma were transfected by X‐tremeGENE reagent.

### 2.2 RNA‐seq and identification of differentially expressed genes

Total RNA was extracted by using the manufacturer's protocol of mirVana miRNA Isolation Kit (Ambion). Agilent 2100 Bioanalyzer (Agilent Technologies) was used to evaluate the RNA integrity. Samples with RNA integrity number ≥ 7 were reserved further analysis. TruSeq Stranded mRNA LT Sample Prep Kit (Illumina) was used to construct libraries according to the manufacturer's instructions. Then, these libraries were sequenced on the Illumina sequencing platform (Illumina HiSeq X Ten) and 150 bp paired‐end reads were generated. To identify differentially expressed genes, pair‐end FASTQ read files were mapped to GRCh38 genomes with hisat2. Differentially expression testing was performed using the R package DESeq2. Only genes with *P*‐value ≤ .05 and fold change ≥2 or ≤1/2 were considered statistically significant.

### 2.3 Proteomics analysis using LC‐MS/MS and identification of differentially expressed proteins

Frozen samples were transferred into 1.5‐mL tubes and lysed with 500 µL digestion buffer supplemented with 1 mmol/L PMSF. Then, the tissue samples were homogenized on the ice and further lysed with sonication. BCA method was used to protein quantification. RP separation was performed on an 1100 HPLC System (Agilent) using an Agilent Zorbax Extend RP column (5 μm, 150 mm × 2.1 mm). Mobile phases A (2% acetonitrile in HPLC water) and B (98% acetonitrile in HPLC water) were used for RP gradient. All analyses were performed by a Q Exactive mass spectrometer (Thermo) equipped with a Nanospray Flex source (Thermo). ProteomeDiscoverer (v.2.2) was used to search all of the Q Exactive raw data thoroughly against the Uniprot database. Database searches were performed with Trypsin digestion specificity. Alkylation on cysteine was considered as fixed modifications in the database searching. For protein quantification method, TMT6‐plex was selected. A global false discovery rate (FDR) was set to 0.01 and protein groups considered for quantification required at least 2 peptides. A *t* test was performed to identify differentially expressed proteins. Only proteins with *P*‐value ≤ .05 and fold change ≥3/2 or ≤2/3 were considered statistically significant.

### 2.4 Construction of glaucoma‐related PPI network

Previous studies demonstrated that differentially expressed genes and proteins might play key roles in the regulatory processes. Furthermore, proteins are coded by genes. Thus, we intersected all differentially expressed genes and proteins. In brief, if a gene and its coding protein were both up‐ or down‐regulated with significant threshold of fold change and p‐value, this gene/protein was considered as glaucoma risk gene/protein. In order to investigate the global function of risk genes, we mapped all these risk genes into the HPRD network and extracted the risk gene associated subnetwork, which named as glaucoma‐related PPI network (Figure 1). Network was viewed by Cytoscape.

### 2.5 Network topological analysis and function enrichment

Multiple topological features of glaucoma‐related PPI network by using the package of ‘igraph’ in R language. For the average path length and cluster coefficient of the network, 1000 random degree‐conserved networks were chosen as control, and the measurement of average path length and cluster coefficient in each random network was counted. *P*‐values were processing by calculating the fraction of the number of average path length/cluster coefficient in random network that is larger than that in the real network. DAVID (https://david.ncifcrf.gov/) and PATHWAX (http://pathwax.sbc.su.se/) were performed for pathway and gene ontology (GO) enrichment analysis.

### 2.6 Western blotting

The total protein of iHTM and GTM_3_ cells was extracted following the instructions of manufactory (GE Healthcare). Protein concentration was detected by bicinchoninic acid (BCA) kit. The extracted proteins were added to loading buffers and then boiled for 10 minutes at 100°C. We used 10% sodium dodecyl sulphate‐polyacrylamide gel electrophoresis (SDS‐PAGE) to isolate proteins at electrophoresis voltages of 80 and 120 V. After electrophoresis, proteins were then transferred to PVDF membranes at voltage of 200 V for 120 minutes. After the membranes were incubated with 5% skim milk at room temperature for 1 hour, CD9 antibody (1:1000, ab92726; Abcam), ITGA4 antibody (1:1000, ab81280; Abcam), p‐PI3K antibody (1:1000, ab182651; Abcam), PI3K antibody (1:1000, ab86714; Abcam), p‐Akt antibody (1:1000, ab38449; Abcam) or Akt antibody (1:500, ab8805; Abcam) was added. The membranes were stored in refrigerator at 4°C overnight. The membranes were washed by TBST for 3 times/10 min and incubated with corresponding second antibody for 2 hours at 37°C. The membrane was washed by TBST for 3 times/10 min, and colour‐developing reagent (ECL) was added to the protein membrane. Imaging was exported by Jena light system. ImageJ software was used to analyse the image. The ratio of the target to GAPDH light density was regarded as the relative concentration of protein expression.

### 2.7 Immunofluorescence staining

Cultured iHTM and GTM_3_ cells on glass coverslips were washed by PBS 3 times and added 4% paraformaldehyde on coverslips for 15 minutes. Then, 0.4% Triton X‐100 was used to permeate the cell membrane for 30 minutes. After 3 times washing, cells were incubated with tubulin antibody (1:100, ab7291; Abcam) and vimentin antibody (1:100, #5741; Cell Signal) overnight at 4°C. Subsequently, cells were incubated with a FITC‐conjugated goat anti‐mouse antibody for 1 hour at 37°C. The cells were then washed by PBS, and DAPI was used to stain nuclei for 5 minutes at 37°C. Immunofluorescence was performed by fluorescence microscope (Nikon).

### 2.8 Flow cytometry with Annexin V‐FITC/propidium iodide (PI) double staining

Cells were washed 3 times by PBS and then processed to a 1 mL single‐cell suspension after 24 hours of transfection. The culture solution was removed by centrifugation (1000 r/min, 4°C, 10 minutes). And then 1 mL suspended cells in PBS were added. The supernatant was discarded by centrifugation (1000 r/min, 4°C, 10 minutes). Next, the cells were resuspended in 200‐μL combined buffer solution. Then, we added 10 μL Annexin V‐FITC and 5 μL PI. The cells were incubated in the dark (37°C, 15 minutes). Our analysis system was a flow cytometer (Becton Dickinson). Cell apoptosis rate was calculated with follows: cell apoptosis rate (%) = (Early apoptotic cells + advanced apoptotic cells)/total cell number × 100%.

### 2.9 Real‐time PCR of CD9 mRNA

Total RNA was extracted from iHTM and GTM3 cells with TRIzol reagent (Invitrogen) according to the manufacturer's instructions. CD9 mRNA was subjected to RT‐PCR with a Real‐Time 7300 PCR apparatus (Applied Biosystems) according to the instruction manual (SYBRII Green Real‐time PCR; Takara). The results were analysed with Applied Biosystems software. The sense and antisense primers for human CD9 and GAPDH were as follows: CD9 (forward: 5′‐ACCTGCTGTTCGGAT‐3′; reverse: 5′‐TCAACGCATAGTGGA‐3′) and GAPDH (forward: 5′‐GGTGGTCTCCTCTGACTTCAACA‐3′; reverse: 5′‐GTTGCTGTAGCCAAATTCGTTGT‐3′).

### 2.10 Statistical analysis

Data are expressed as mean ± standard deviation (SD). R was used for statistical analysis, and significance was evaluated by U test. *P*‐values < .05 were considered statistically significant.

## 3 RESULT

### 3.1 Identification of differentially expressed genes

**Figure 2:**
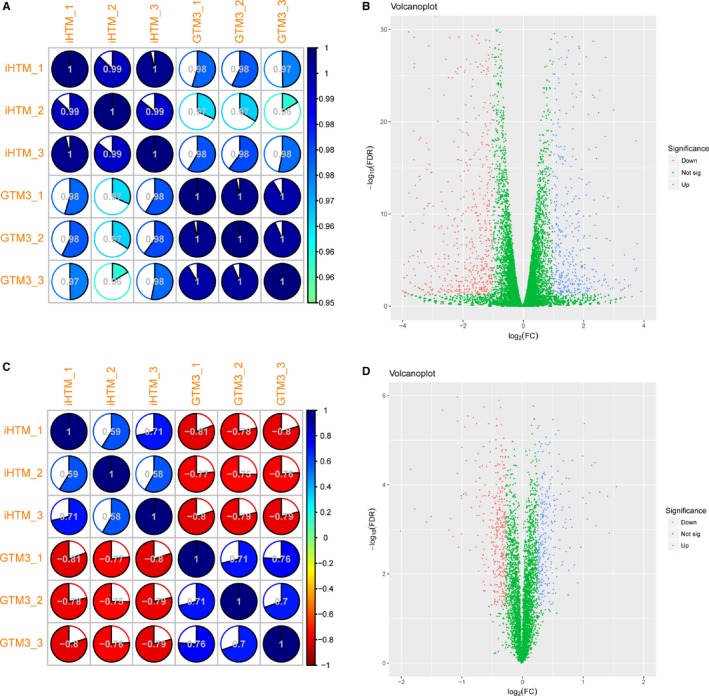
Correlation and differential analysis of high‐throughput transcriptome and proteome data. A, Correlation plot of transcriptome data. B, Volcano plot of transcriptome data. C, Correlation plot of proteome data. D, Volcano plot of proteome data

Differentially expressed genes are always considered as high‐risk molecules in disease development. In order to identify more crucial regulators in the pathological processes of glaucoma, high‐throughput transcriptome and proteome analysis was performed to iHTM (3 samples) and GTM_3_ (3 samples) cells. After calling the expression of transcriptome and proteome data, we performed correlation analysis to these samples. High correlations in biological duplications were shown with the Pearson correlation coefficients > .9 for transcriptome data and >.5 for proteome data (Figure 2A,C), suggesting that our data were stable and credible. Next, differential expression analysis was performed to transcriptome and proteome data, respectively. As for the transcriptome data, DEseq2 was performed to identify differentially expressed genes with the strict threshold of fold change ≥2 or ≤1/2 or *P*‐value < .05. In addition, to confirm the expression of the differentially expressed genes identified by the RNA‐seq, we performed low‐throughput PCR to detect gene expression. Results showed that top changed genes identified from RNA‐seq showed same expression trend in PCR and RNA‐seq results (Figure S1). As for the proteome data, *t* test and fold change analysis were performed to identify differentially expressed proteins with the strict threshold of fold change >1.2 or <5/6 or *P*‐value < .05. As a result, a large number of genes and proteins showed an up or down tendency between iHTM and GTM_3_ (Figure 2B,D), implying that genes might regulate the biological processes of glaucoma in a synergistic way.

**Figure 3:**
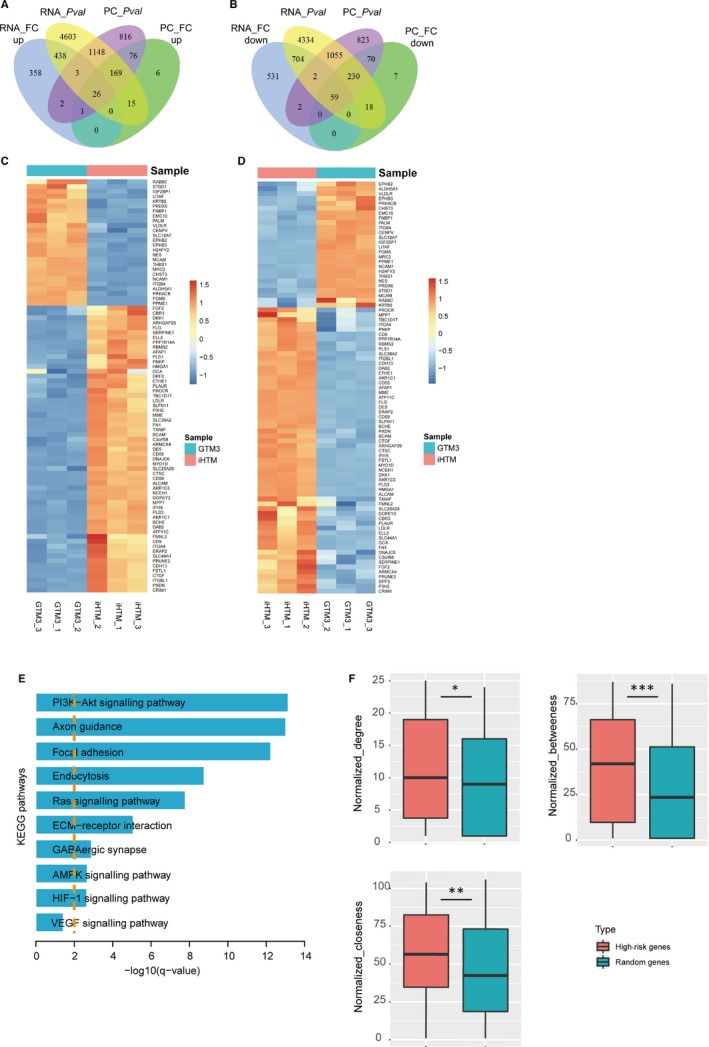
Identification of differentially expressed genes and proteins. A, The Venn plot of differentially expressed genes and proteins (Up‐regulated). RNA_FC up: up‐regulated genes based on fold change; RNA_Pval: differentially expressed genes based on *P*‐value; PC_FC up: up‐regulated proteins based on fold change; PC_Pval: differentially expressed proteins based on *P*‐value. B, The Venn plot of differentially expressed genes and proteins (Down‐regulated). RNA_FC down: down‐regulated genes based on fold change; RNA_Pval: differentially expressed genes based on *P*‐value; PC_FC down: down‐regulated proteins based on fold change; PC_Pval: differentially expressed proteins based on *P*‐value. (C‐D) Heat map of high‐risk factors in RNA levels (C) and protein levels (D). E, Pathway enrichment of high‐risk factors. F, Topology features (degree, closeness and betweenness) comparison between high‐risk factors and random genes in HPRD PPI network. * represents *P* < .05, ** represents *P* < .01 and *** represents *P* < .001

Proteins were coded by genes in ribosome. Post‐transcriptional modification and post‐translational modification can lead to the inconformity between expression levels of genes and corresponding proteins. Thus, to identity more accurate result and reduce false positives, we combined both transcriptome and proteome differential expression results and took an intersection; results showed that a fraction of genes and corresponding proteins were both identified as differentially expressed factors (Figure 3A,B). In brief, we only reserved the genes that were both differentially expressed in RNA and protein levels as the high‐risk genes. After this filter, 26 up‐regulated genes/proteins and 59 down‐regulated genes/proteins were identified (Figure 3C,D), including the famous molecule of glaucoma, such as fibronectin (FN1) and connective tissue growth factor (CTGF). FN1 and CTGF were both down‐regulated in glaucoma cells (GTM_3_ cells). FN1 is a biomarker of TM cells. Filla et al found that fibronectin fibrils are the major component of extracellular matrix of TM cells. Disruption of fibronectin fibrils can disrupt the incorporation of type IV collagen, laminin and fibrillin into the extracellular matrix, whereas abnormal deposition of extracellular matrix can lead to glaucoma.^15^ Furthermore, CTGF was also demonstrated as the matricellular proteins in glaucoma, which plays a role in fibrosis and increased extracellular matrix deposition.^16^ CTGF might be relevant for the development of elevated IOP, which is considered as a high‐risk factor in glaucoma pathogenesis.^17^ These results indicated high‐risk genes were key regulators in pathogenesis of glaucoma.

Genes always disrupt biological function by involving downstream signalling pathways. Thus, we performed network‐based pathway enrichment analysis to high‐risk gene sets. Result showed that all high‐risk genes were high related to some conditional pathways, such as PI3K‐Akt, focal adhesion, endocytosis and ECM‐receptor interaction (Figure 3E), all of which were demonstrated to play crucial roles in glaucoma. Li et al found that MALAT1 could suppress apoptosis activity of retinal ganglion cells through activating of the PI3K/Akt signalling pathway in glaucoma. Latanoprost is a powerful antiglaucoma drug with ocular neuroprotective and hypotensive effects, which exerts functions by promoting neurite outgrowth through a prostaglandin F receptor‐mediated modulation of the PI3K‐Akt‐mTOR signalling pathway.^18^ Our previous study demonstrated that sustained pressure elevation could directly induce trabecular meshwork cell damage by injuring zonula occludens‐1, cytoskeleton and foal adhesions.^19^ Wu et al demonstrated that knockout of Caveolin‐1 can reduce adhesion with higher extracellular matrix‐degrading enzyme expression, but increase endocytosis and autophagy activities, indicating that Caveolin‐1 might participate in the regulatory processes of endocytosis, adhesion and autophagy in human TM cells.^20^

### 3.2 Construction of glaucoma‐related PPI network

**Figure 4:**
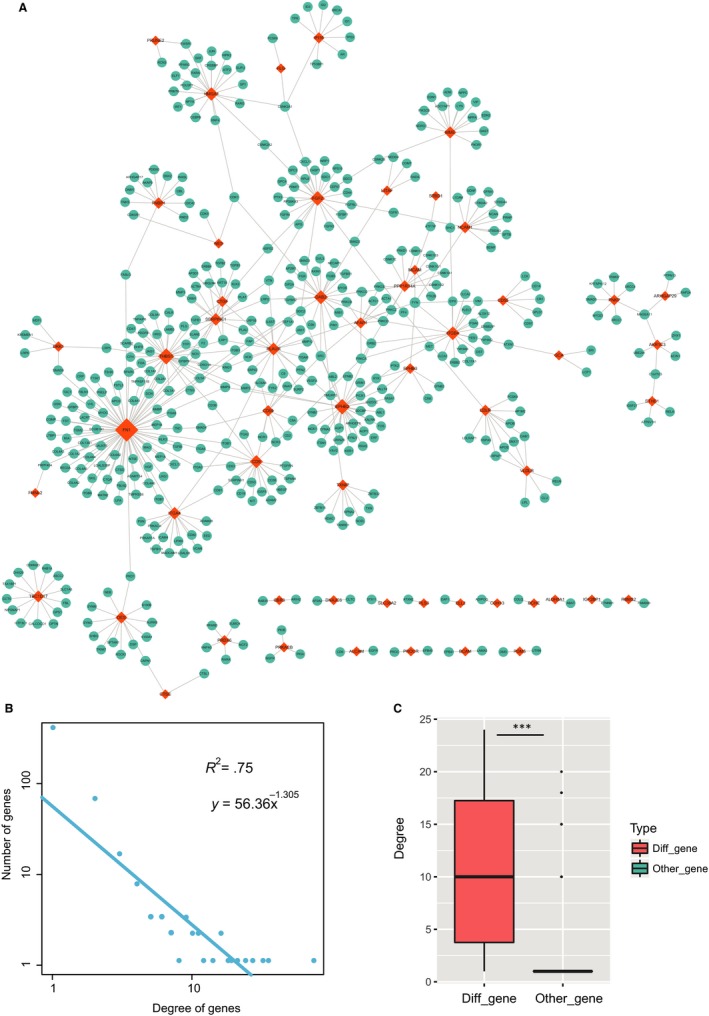
Global view and topology features of glaucoma‐related PPI network. A, The visualization of glaucoma‐related PPI network. Orange nodes represented high‐risk factors and blue nodes represented their partners in PPI. Node size represented degrees. B, Degree distributions of the network. All degrees followed a power‐law distribution. C, Degree comparison of differentially expressed genes and non‐differentially expressed genes. *** represents *P* < .001

We mapped all high‐risk genes to the PPI network of Human Protein Reference Database (http://www.hprd.org). Results showed that all the genes were more crucial than random genes (Figure 3F), suggesting that these high‐risk genes can regulate multiple functions. To further investigate the function of high‐risk genes in a global view, a glaucoma‐related PPI network was proposed and constructed. Firstly, we downloaded human experimentally validated PPI network from Human Protein Reference Database and mapped all high‐risk genes into PPI network. Secondly, we extracted all the interaction partners of risk genes in PPI and merged them into the glaucoma‐related PPI network. As a result, we extracted 522 interactions from PPI network, including 483 nodes (Figure 4A).

### 3.3 Topological feature analysis of glaucoma‐related PPI network

**Figure 5:**
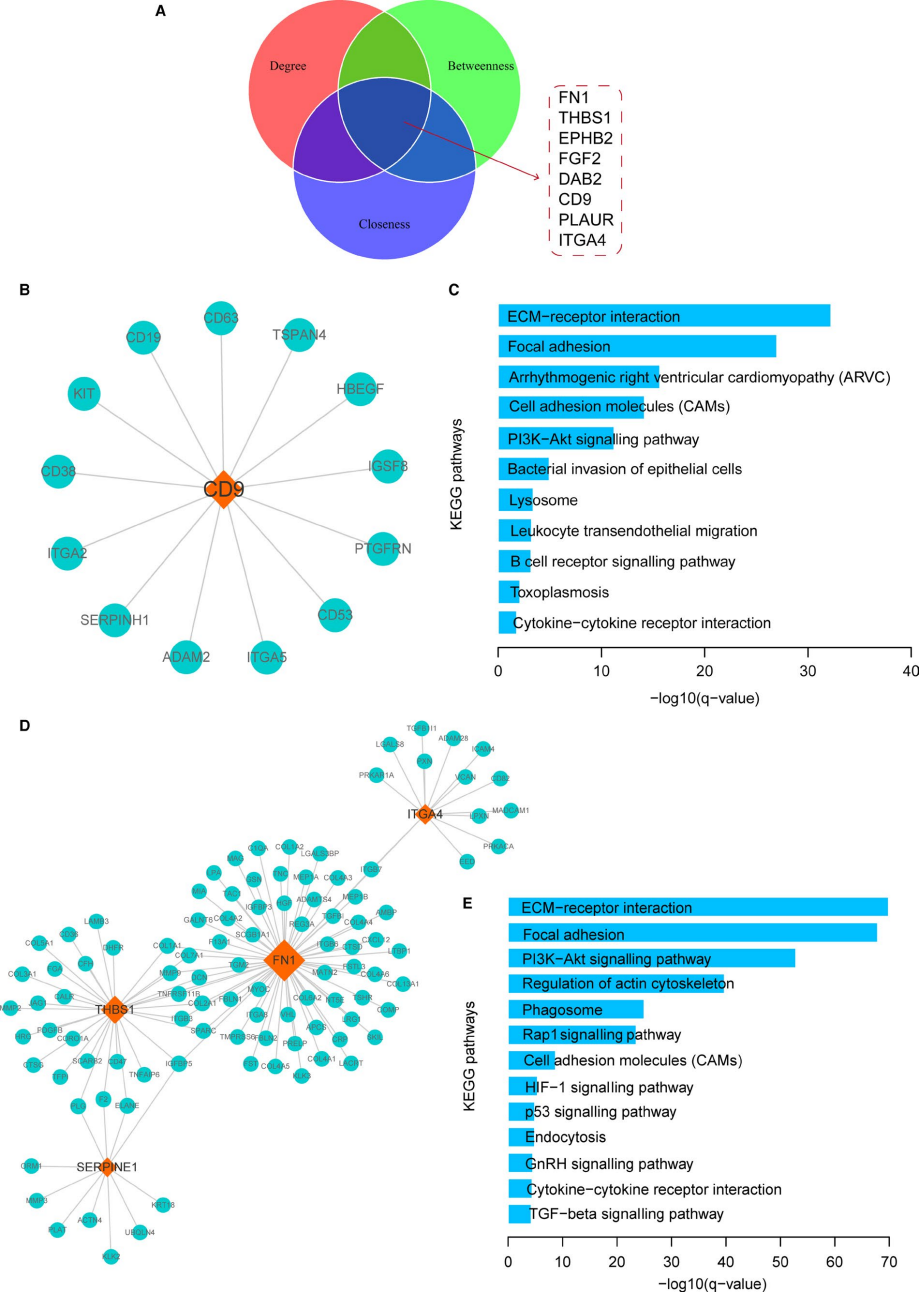
Module analysis of ODLMN. A, The distributions of top 20 nodes of degree, betweenness and closeness. B, The view of module 1. C, Pathway enrichment analysis for mRNAs in module 1. D, The view of module 2. E, Pathway enrichment analysis for mRNAs in module 2

After constructing the glaucoma‐related PPI network, topological analysis was performed to the network. Previous studies found that biological network‐based analysis could identify novel and key regulators in regulating biological processes. Firstly, degree analysis was performed to the network. Results showed that all degrees of nodes followed a power‐law distribution (Figure 4B), indicating that the network was scale‐free, a small subnet of hub nodes linked many interacting partners, similar as most of biological networks. Differentially expressed genes occupied the central core of network (Figure 4C), suggesting that differentially expressed genes might play crucial roles in the physiological processes of glaucoma. In addition, studies demonstrated that genes with central topological features in biological network always played crucial regulatory role in biology. Thus, topology features of degree, betweenness and closeness were calculated for nodes in network, respectively. We then selected top 20 crucial nodes of each topological feature. Interestingly, we found 8 genes (FN1, THBS1, EPHB2, FGF2, DAB2, CD9, PLAUR and ITGA4) were crucial (Figure 5A), suggesting that these genes might function as key factors in the pathogenesis of glaucoma.

### 3.4 Module analysis of glaucoma‐related PPI network

Large number of studies has showed that genes prefer to function in modules. Thus, we performed module analysis for the network using FAG‐EC algorithm that was embedded in cytoscape. As a result, two functional modules that located nearby in network were identified. We then extracted the corresponding gene interactions of the two dense modules. Module 1 was comprised by 14 genes and was regulated by CD9 (Figure 5B). Pathway analysis results showed these genes were enriched in some glaucoma‐related pathways were enriched, such as ECM‐receptor interaction, focal adhesion, cell adhesion and PI3K‐Akt signalling pathway (Figure 5C).

Module 2 was a complex module that included 106 genes and was drived by 4 genes (FN1, ITGA4, THBS1 and SERPINE1) (Figure 5D). We also enriched pathways for module 2 (Figure 5E). Results showed that some glaucoma‐related pathways were enriched, such as ECM‐receptor interaction, PI3K‐Akt signalling pathway, regulation of actin cytoskeleton, HIF‐1 signalling pathway and endocytosis. Dysfunction of actin cytoskeleton can lead to the dysfunction of glaucoma. Junglas et al found that the effects of CTGF on IOP appear to be caused by a modification of the TM actin cytoskeleton.^21^ Moreover, HIF‐1α protein was up‐regulated in the retina following elevation of IOP.^22^ These results indicated that crucial regulators could function by implicating in close gene modules, regulating downstream pathway signal of glaucoma.

### 3.5 CD9 is a crucial regulator and is down‐regulated in glaucoma

**Figure 6:**
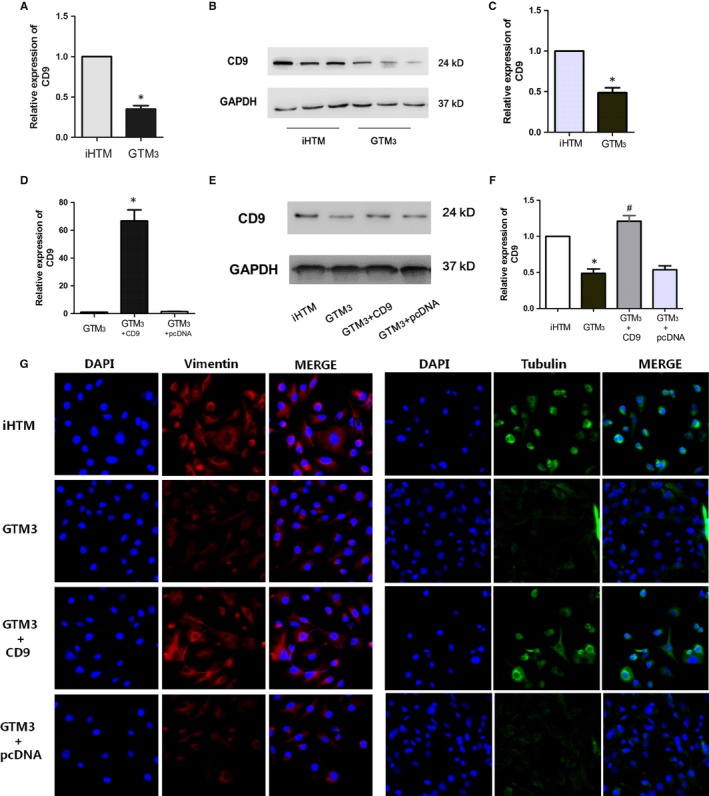
CD9 is the candidate regulator in glaucoma and overexpression of CD9 maintained TM cell identity. A, Real‐time PCR result of CD9 mRNA in iHTM and GTM_3_ cells. n = 6. (B‐C) Protein expression of CD9 in iHTM and GTM_3_ cells. n = 6. D, Real‐time PCR result of CD9 mRNA in GTM_3_, GTM_3_ + CD9 and GTM_3_ + pcDNA3.1 groups. n = 6. (E‐F) Protein expression of CD9 in iHTM, GTM_3_, GTM_3_ + CD9 and GTM_3_ + pcDNA3.1 groups. n = 6. *P < 0.05 vs iHTM group, ^#^ *P* < .05 vs GTM_3_ group. G, Immunostaining of Vimentin and Tubulin in iHTM, GTM_3_, GTM_3_ + CD9 and GTM_3_ + pcDNA3.1 groups

**Figure 7:**
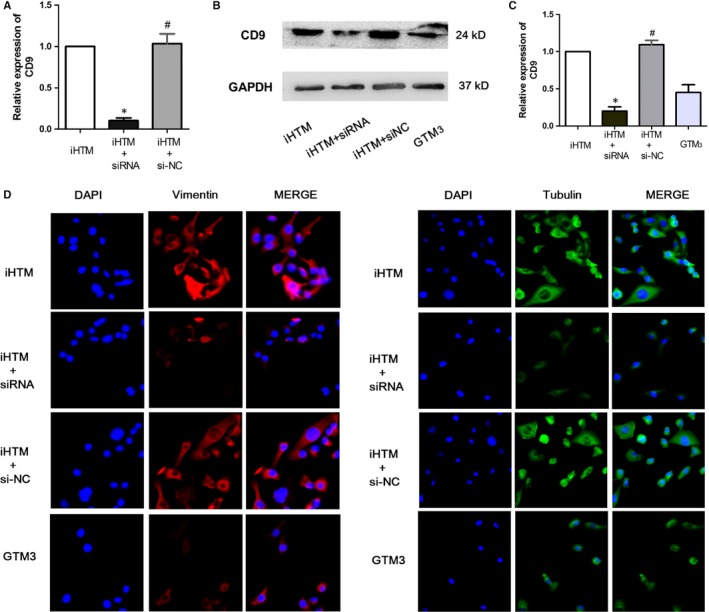
Knock‐down of CD9 changed TM cell identity. A, Real‐time PCR result of CD9 mRNA in iHTM, iHTM + siRNA, iHTM + si‐NC and GTM_3_ groups. n = 6. (B‐C) Protein expression of CD9 in iHTM, iHTM + siRNA, iHTM + si‐NC and GTM_3_ groups. n = 6. *P < .05 vs iHTM group, ^#^ *P* < .05 vs iHTM + siRNA group. D, Immunostaining of Vimentin and Tubulin in iHTM, iHTM + siRNA, iHTM + si‐NC and GTM_3_ groups

Based on the results of previous steps, some crucial genes were identified. As we all known that, FN1 is a discovered gene that participated in the regulatory processes of extracellular matrix in glaucoma. And some studies found that FN1 was involved in the pathway of PI3K‐Akt and focal adhesion. Pathway enrichment result is consistent to the mechanism. Next, we want to identify and validate novel regulators that function in glaucoma. In module 1, CD9 is the core node and controls all the functions of modules. Furthermore, module 1 is closed to module 2 and is connected by multiple gene crosstalks, implying module 1 might play the similar role in glaucoma. Thus, we detected the expression levels of CD9 in iHTM cells and GTM_3_ cells on RNA level and protein level, and results showed that CD9 was both significantly decreased in glaucoma cells (Figure 6A‐C), which suggested that CD9 might exert a protective role in glaucoma. Thus, we then constructed a plasmid to overexpress CD9, and results showed that CD9 is overexpressed successfully by transfection (Figure 6D‐F). Previous studies found that vimentin is a cell identity marker of TM cells and glaucoma development is followed with vimentin hydrolysis. Furthermore, studies found that high intraocular pressure can lead a decrease in the TM cell cytoskeleton protein, which is a primary risk of glaucoma. Thus, we performed immunofluorescence staining of two markers (tubulin and vimentin) to investigate the functional role of CD9 for maintaining normal TM cell identity, and results showed that overexpression of CD9 can reverse the expression of tubulin and vimentin, maintaining the normal TM cell identity (Figure 6G). For further validation of the functional role of CD9 in pathogenesis of glaucomatous changes, we also used a siRNA to knock‐down CD9 in iHTM cells. Real‐time PCR and Western blot results indicated that CD9 was inhibited successfully (Figure 7A‐C). Moreover, knock‐down of CD9 can lead to the decrease expression of tubulin and vimentin of TM cells, implying decreased CD9 can change TM cell identity (Figure 7D).

### 3.6 CD9/ITGA4/PI3K‐Akt axis mediated apoptosis to regulate glaucoma

**Figure 8:**
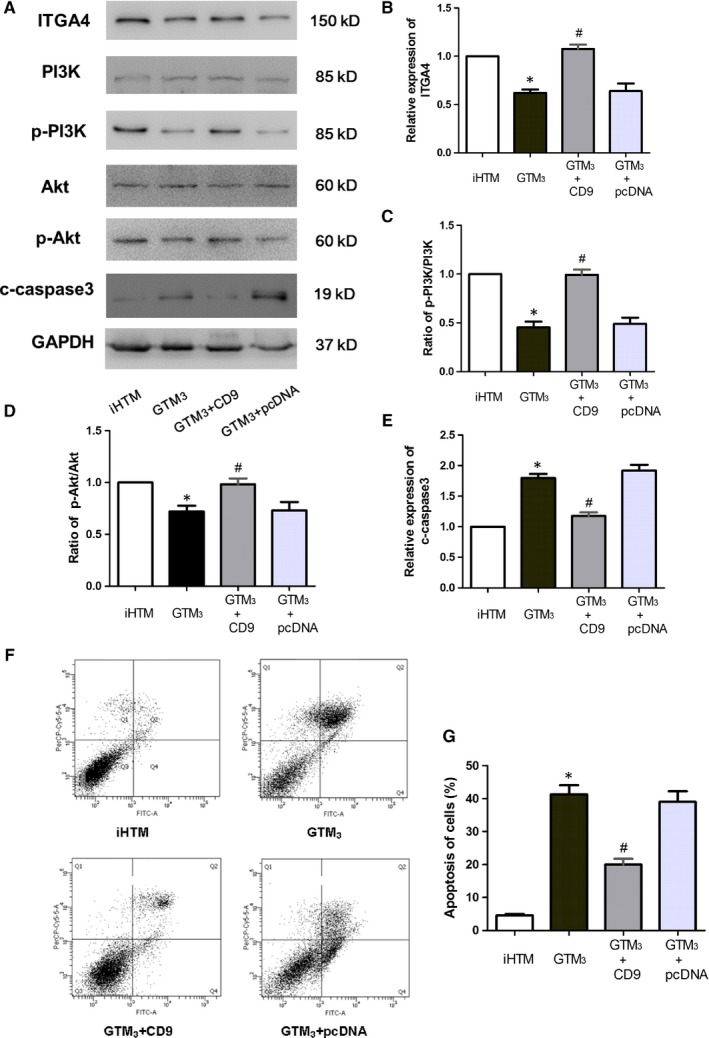
CD9/ITGA4/PI3K‐Akt axis mediates trabecular meshwork cell apoptosis in human glaucoma. (A,B) Protein expression of ITGA4 in GTM_3_, GTM_3_ + CD9 and GTM_3_ + pcDNA3.1 groups. n = 6. (A,C) Protein expression of p‐PI3K in GTM_3_, GTM_3_ + CD9 and GTM_3_ + pcDNA3.1 groups. n = 6. (A,D) Protein expression of p‐Akt in GTM_3_, GTM_3_ + CD9 and GTM_3_ + pcDNA3.1 groups. n = 6. (A,E) Protein expression of clevage caspase3 in GTM_3_, GTM_3_ + CD9 and GTM_3_ + pcDNA3.1 groups. n = 6. *P < .05 vs iHTM group, ^#^ *P* < .05 vs GTM_3_ group. (F‐G) FCAS results of cells from iHTM, GTM_3_, GTM_3_ + CD9 and GTM_3_ + pcDNA3.1 groups. n = 3. *P < .05 vs iHTM group, ^#^ *P* < .05 vs GTM_3_ group

**Figure 9:**
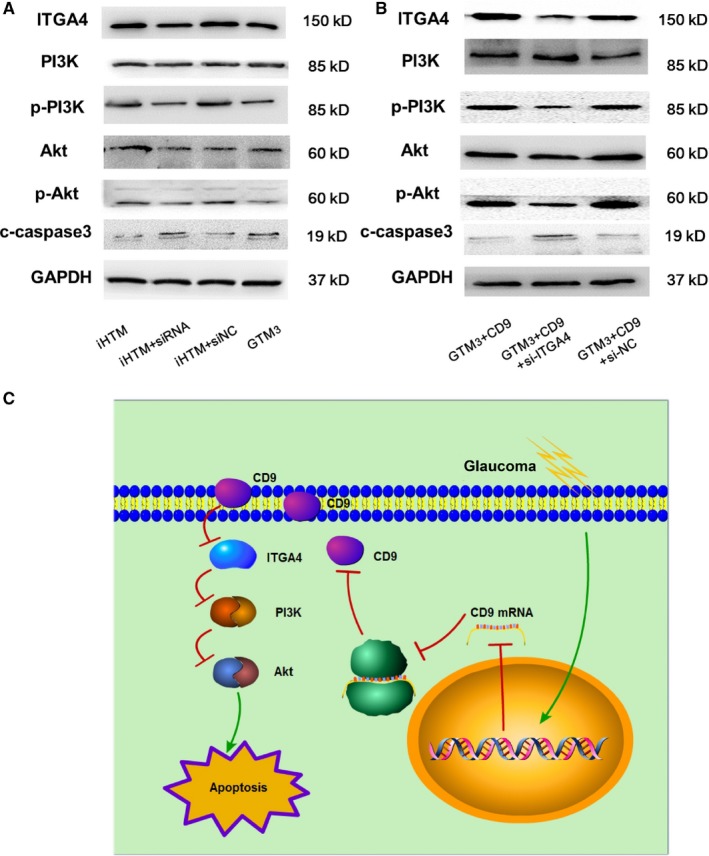
CD9/ITGA4/PI3K‐Akt axis participating in regulating glaucoma. A, Protein expression of ITGA4, PI3K, Akt and caspase‐3 in iHTM, iHTM + siRNA, iHTM + si‐NC and GTM_3_ groups. B, Protein expression of ITGA4, PI3K, Akt and caspase‐3 in GTM_3_ + CD9, GTM_3_ + CD9+si‐ITGA4 and GTM_3_ + CD9+si‐NC groups. C, Mimic mechanism pathway of CD9/ITGA4/PI3K‐Akt axis mediates trabecular meshwork cell apoptosis in human glaucoma

Next, we want to investigate the molecular mechanism of CD9 in regulating glaucoma. In network, CD9 is closely linked to FN1 via integrin proteins and CD9 has an integrin‐binding function. Thus, we hypothesized that CD9 might regulate upstream integrin proteins to exert functions. ITGA4 is also a high‐risk gene in glaucoma and shows a down‐regulated tendency in glaucoma. Furthermore, ITGA4 is the upstream gene of PI3K‐Akt pathway. Overexpression of CD9 leads to a significant increase of ITGA4, PI3K and Akt and knock‐down of CD9 significantly decreased the expression of ITGA4, PI3K and Akt, which suggested that CD9 can regulate PI3K‐Akt activity via ITGA4 (Figure 8A‐D and Figure 9A). Previous studies found that PI3K activity was negatively correlated with apoptosis activity, and we also detected the activity of apoptosis. Results showed that overexpression of CD9 can lead to the decreased apoptosis (Figure 8A,E,F and G). However, knock‐down of CD9 in iHTM can lead to elevated apoptosis activity (Figure 9A). Additionally, we also performed rescue experiment of knock‐down ITGA4 with the CD9 overexpression to confirm whether the reduction in cell apoptosis by CD9 can be reversed. As a result, knock‐down of ITGA4 can maintain the cell apoptosis activity (Figure 9B), indicating ITGA4 is the downstream mediator of CD9. Moreover, suppression of apoptosis can maintain the function of trabecular meshwork cells. All these results showed that CD9/ITGA4/PI3K‐Akt axis can mediate apoptosis activity to attenuate glaucoma.

## 4 DISCUSSION

Glaucoma and specially POAG have been the leading cause of blindness worldwide, which poses a new challenge to the public health.^23^ Furthermore, glaucoma has the characteristic of high incidence, high blindness and irreversibility. Thus, it is urgent to uncover the molecular mechanism of glaucoma. High IOP is the major risk factor of glaucoma, and repression of IOP is the important treatment of glaucoma in clinic.^24^, ^25^ Maintenance of IOP is depended on the dynamic balance of aqueous humour generation and out‐flow. An elevated IOP is caused by excessive resistance to the out‐flow of the aqueous humour. The regulatory mechanism of aqueous humour out‐flow is unknown.^26^ Most of all, trabecular meshwork is the key part of aqueous humour out‐flow. Trabecular meshwork is composed by extracellular matrix, collagen bundle and trabecular meshwork cells. Dysfunction of trabecular meshwork cells can lead to the lesion of trabecular meshwork. Trabecular meshwork cells are important for aqueous humour out‐flow and maintenance of IOP because of its function of phagocytosis, contraction and metabolism regulation. Previous studies found that autophagic dysregulation in glaucomatous trabecular meshwork cells had obvious pathologic changes in POAG.^27^ Thus, in this study, we focused on identifying novel molecules that function in glaucoma in trabecular meshwork cells.

Previous studies revealed some crucial regulators in multiple diseases by combining multi‐omics data. Thus, to identify more accurate results, we performed high‐throughput transcriptome and proteome analysis to iHTM and GTM_3_ cells, respectively. Firstly, we calculated the differentially expressed genes and proteins and selected the genes/proteins that were both differentially expressed in transcriptome and proteome data as the high‐risk factors. Results showed that 26 up‐regulated genes/proteins and 59 down‐regulated genes/proteins were identified, including some known factors of glaucoma. To investigate the function of these genes in a global view, we constructed glaucoma‐related PPI network by mapping high‐risk genes into the HPRD PPI network. In this scale‐free network, we found that 8 genes occupied the central topology features of degree, closeness and betweenness. In addition, we performed module analysis to the network and two close modules were identified. Module 1 was drived by CD9 and module 2 was drived by FN1, ITGA4, THBS1 and SERPINE1. These modules were highly related to crucial downstream pathways, such as ECM‐receptor interactions, focal adhesion and PI3K‐Akt. Moreover, in module 2, FN1 is the famous molecule in pathogenesis of glaucoma. And in module 2, CD9 is linked closely to FN1 through integrin family proteins. Thus, we thought that CD9 might be a candidate regulator in glaucoma.

CD9 locates on cell surface and is a known investigated molecule that function in cell migration and adhesion.^28^ In cancer field, previous studies found that CD9 is preferentially expressed in glioma stem cells (GSCs) of human glioblastoma multiforme tumours. Disruption of CD9 can significantly inhibit the self‐renewal and promoted the differentiation of GSCs. CD9 disruption markedly reduced gp130 protein levels and STAT3 activating phosphorylation in GSCs.^29^ Moreover, studies found that CD9 is high associated with PI3K activity. Wang et al found that CD9 decreased the phosphorylation of epidermal growth factor receptor (EGFR), which leads to the changed activity of PI3K/Akt and MAPK/Erk. CD9 attenuated EGFR signalling of PI3K/Akt and MAPK/Erk, which was associated with cell growth and proliferation.^30^ Western blot results showed that CD9 is down‐regulated in glaucoma cells. And we firstly demonstrated that disruption of CD9 expression in TM cells could change the TM cell identity. These results suggested that CD9 is a key regulator in pathogenesis of glaucoma.

Interestingly, in our filter results, we found 2 genes (CD9 and ITGA4) had a great potential to function in glaucoma. CD9 has the biological function of integrin binding,^31^, ^32^ and CD9 linked to FN1 through multiple integrin family proteins. Thus, whether CD9 can participate in glaucoma via ITGA4 and downstream pathways is proposed by our study. To demonstrate this regulatory mechanism, we performed biological experiment in normal and glaucomatous cells. Results showed that ITGA4 is also shown a down‐regulated tendency in glaucoma.

Next, we identified the downstream regulatory axis of CD9 and ITGA4. Studies found that ITGA4 is the upstream genes of PI3K‐Akt pathway.^33^, ^34^ Id1 and ITGA4 expression were increased in endothelial progenitor cells (EPCs) from ovarian cancer patients compared with those obtained from healthy samples. Knock‐down of Id1 substantially reduced EPCs function and ITGA4 expression. Importantly, inhibition of PI3K/Akt inhibited Id1 and ITGA4 expression, resulting in the decreasing biological function of EPCs.^35^ Based on the pathway enrichment results, we can draw the conclusion that PI3K‐Akt was the potential downstream pathways in glaucoma. We then overexpressed CD9 by plasmid in GTM_3_ cells, and results showed that the phosphorylated PI3K and Akt were both elevated significantly. A large number of studies found that PI3K activity was negatively correlated apoptosis activity.^36^, ^37^ Suppression of apoptosis can maintain the function of trabecular meshwork cells.^38^, ^39^ Thus, we detected the activity of apoptosis of cells. Results showed that glaucoma cells have increased apoptosis activity than control cells. Overexpression of CD9 can decrease the apoptosis activity of glaucoma cells, which was benefit for glaucoma treatment. Furthermore, we also knocked down of CD9 in iHTM cells, results showed that ITGA4, phosphorylated PI3K and Akt were both significantly decreased. And knocked down of ITGA4, the expression of phosphorylated PI3K and Akt was rescued. These results indicated that CD9/ITGA4/PI3K‐Akt axis mediates trabecular meshwork cell apoptosis in human glaucoma.

Overexpression of CD9 down‐regulates Wnt1 and Wnt signal pathways, which play important roles in TM function and regulation of intraocular pressure (IOP). It is known that IOP elevation can be blocked by the activation of the downstream canonical Wnt pathway. Thus, we also analysed the significant changed genes of Wnt pathway in glaucoma. Results showed that 8 genes were significantly changed, 6 of which were down‐regulated in glaucoma, such as WNT7B and DKK1 (Table S1). WNT7B is the most differential gene of Wnt pathway. These down‐regulated genes indicated the decreased Wnt pathway activity in glaucoma, which can lead to the decreased cell proliferation and increased cell apoptosis.

However, our study also has a limitation. We used immortalized TM cell lines to investigate the functional mechanism of glaucoma. We performed bioinformatics analysis to compare the marker genes of primary TM cells and other genes. Results showed that marker genes of primary TM cells were high expressed in the immortalized TM cell lines. Furthermore, we also performed marker gene immunofluorescence staining for immortalized TM cell lines. All these results indicated immortalized TM cell lines have the TM cell identity. Nevertheless, primary TM cells are recommended to be used for glaucoma research.

In conclusion, we performed a systematic analysis to identify novel regulators that function in pathological processes of glaucoma. A regulatory axis of CD9/ITGA4/PI3K/Akt/Apoptosis was identified (Figure 9C). In glaucoma, overexpression of CD9 can activate expression of ITGA4, phosphorylated PI3K, and Akt, which lead to the inactivation of apoptosis. All these results provide a novel molecular therapy of glaucoma.

## CONFLICT OF INTEREST

The authors declare that they have no conflicts of interest to disclose.

## AUTHOR CONTRIBUTIONS

Junwei Yan and Xuejiao Yang designed this project and conducted the experiments, Xuefei Jiao and Yunqing Chen processed the data, Xian Yang and Mingjin Guo directed the research, and Lu Zhan and Wenshi Chen wrote the manuscript.

## Data Availability

The data used to support the findings of this study are available from the corresponding author upon request.
