# Supplementary figures and images for: Integrative transcriptomic and proteomic analysis reveals CD9/ITGA4/PI3K‐Akt axis mediates trabecular meshwork cell apoptosis in human glaucoma
Source: J Cell Mol Med. 2019 Nov 3;24(1):814–29. doi: 10.1111/jcmm.14792 (PMC6933396; doi:10.1111/jcmm.14792)

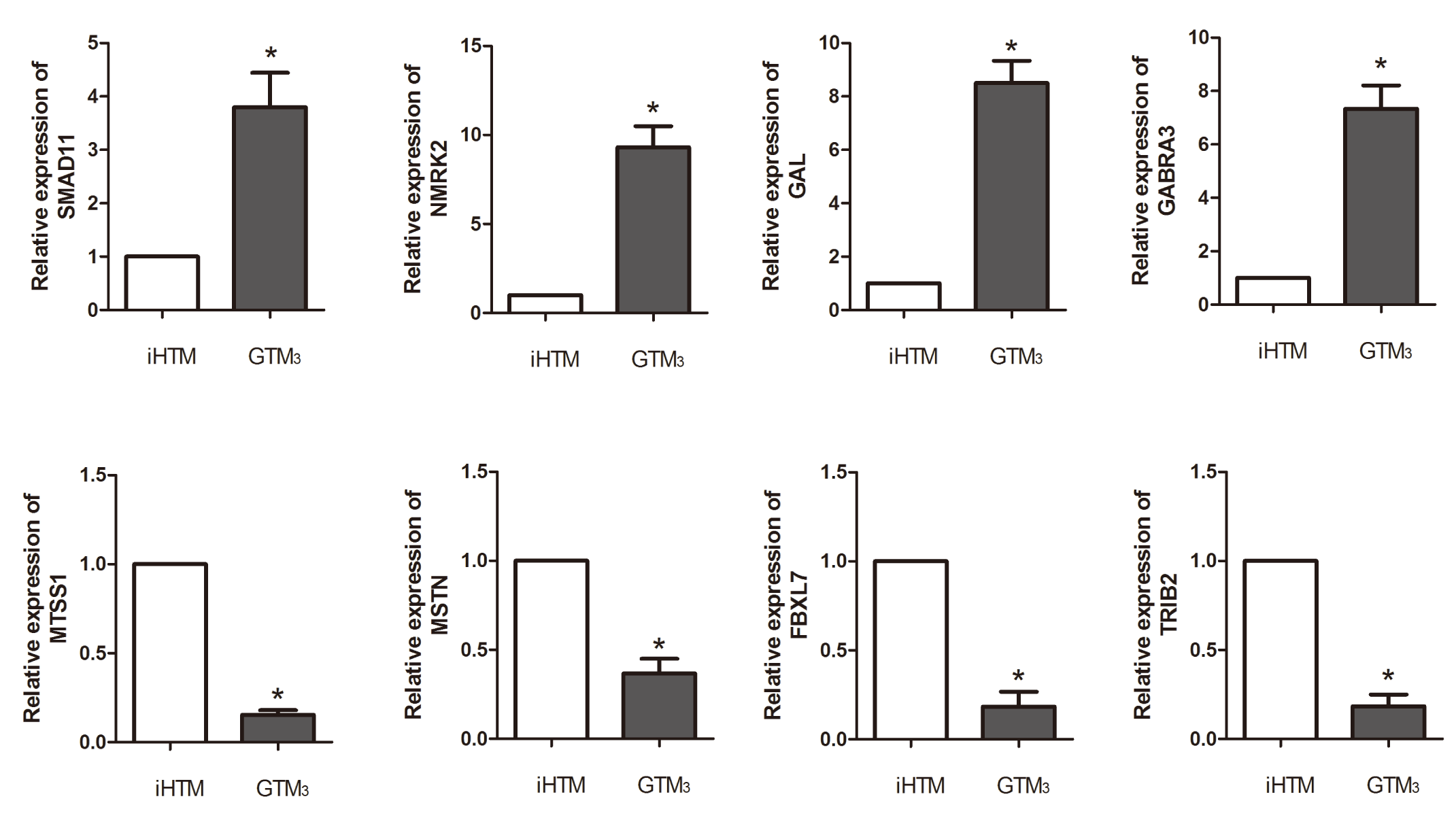

Supplement: Supplementary file 1 [file JCMM-24-814-s001.tif]

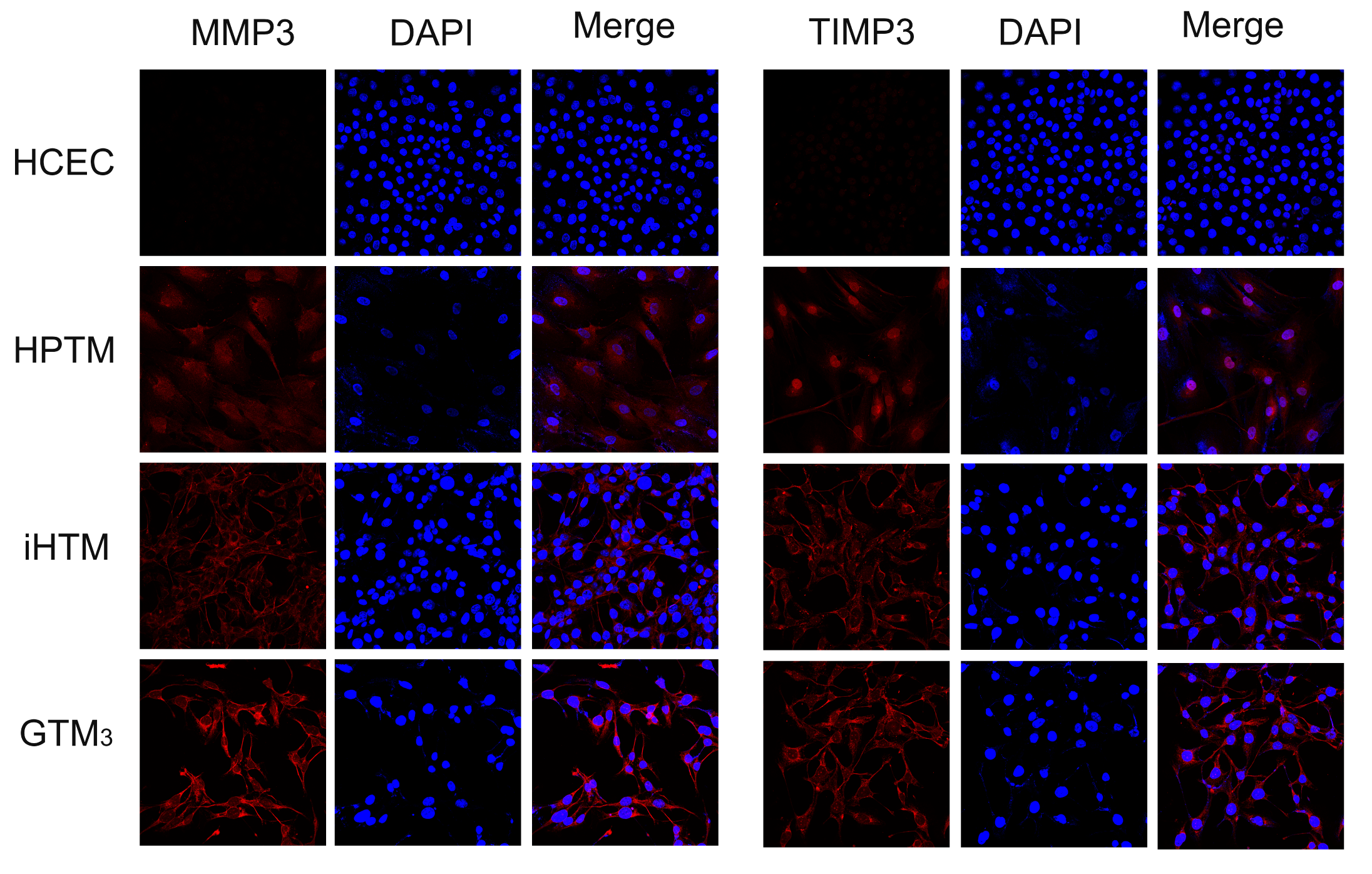

Supplement: Supplementary file 2 [file JCMM-24-814-s002.tif]
